# Prognostic impact of intraoperative peritoneal cytology in interval debulking surgery for pelvic high‐grade serous carcinoma

**DOI:** 10.1002/cam4.2377

**Published:** 2019-06-26

**Authors:** Naoki Kojima, Hiroshi Yoshida, Ikumi Kuno, Takashi Uehara, Masaya Uno, Mitsuya Ishikawa, Tomoyasu Kato

**Affiliations:** ^1^ Department of Pathology and Clinical Laboratories National Cancer Center Hospital Tokyo Japan; ^2^ Department of Gynecology National Cancer Center Hospital Tokyo Japan

**Keywords:** high‐grade serous carcinoma, interval debulking surgery, no macroscopic residual disease, ovarian cancer, peritoneal washing cytology

## Abstract

**Background and Objectives:**

The aim of this study was to determine whether peritoneal washing cytology (PWC) during interval debulking surgery (IDS) could predict the prognosis of patients with pelvic high‐grade serous carcinoma (HGSC) achieving R0 status.

**Methods:**

Between January 2007 and May 2018, 110 patients with ovarian/tubal/primary peritoneal HGSC received platinum‐based neo‐adjuvant chemotherapy, followed by IDS at National Cancer Center Hospital, Japan. All the patients achieved R0 debulking status, defined as no macroscopic residual tumor in the peritoneal cavity at the completion of IDS. PWC was performed before debulking during IDS. The survival outcomes were compared between the PWC‐positive and PWC‐negative groups.

**Results:**

The median progression free survival (PFS) for the entire cohort was 17 months (range, 5‐133 months). The median PFS for the PWC‐positive group was significantly shorter than that of the PWC‐negative group (16 vs 19 months, HR 2.04, 95% CI 1.22‐3.41, *P*‐value < 0.01). Increased risk of progression was observed on both univariate and multivariate analyses, including age and FIGO stage (HR 2.28; 95% CI 1.35‐3.84, *P *<* *0.01).

**Conclusions:**

The positive PWC during IDS was found to predict earlier disease recurrence in patients with pelvic HGSC achieving R0 status. As performing PWC during IDS becomes standard practice, prospective validation should be conducted in the future.

## 1 INTRODUCTION

Ovarian cancer is the eighth most common cause of cancer‐related deaths in women worldwide.^1^ Ovarian cancer shows the highest mortality of all gynecological cancers. High‐grade serous carcinoma (HGSC) of tubo‐ovarian/peritoneal origin is the most common subtype and accounts for 70%‐80% of ovarian cancer deaths, and survival outcome has not dramatically improved for several decades.^2^, ^3^ The poor prognosis of HGSC is mainly due to the diagnosis at an advanced stage of disease and therapy‐resistant disease relapses that occur in the majority of patients following initial treatment with cytoreductive surgery and platinum‐containing chemotherapy.^3^

Recently, the main treatment strategies of patients with advanced HGSC has been composed of primary debulking surgery followed by platinum‐based chemotherapy or interval debulking surgery (IDS) after neoadjuvant chemotherapy (NACT).^4^, ^5^ Achieving minimal to no macroscopic residual disease after surgery for HGSC has been established as a validated prognostic factor.^6^ A meta‐analysis of 6885 patients collected from 81 cohorts of advanced ovarian cancer patients who underwent surgery followed by chemotherapy elucidated that the median survival time was prolonged by 5.5% for every 10% increase in optimal cytoreduction.^7^ To date, optimal debulking is regarded as <1 cm of residual tumor at the completion of surgery; moreover, previous studies have revealed that no macroscopic residual tumor (R0) status can provide significantly prolonged survival time.^8^ However, the clinical course of patients achieving R0 status substantially varies, which may reflect the varying extents of microscopic residual disease. To date, it remains difficult to predict the prognosis of patients achieving R0 status by assessing microscopic residual disease.

Peritoneal washing cytology (PWC) is a sensitive indicator of ovarian surface involvement and peritoneal dissemination by ovarian tumors.^9^, ^10^ PWC is not a novel technique; however, it is simple, less invasive, inexpensive, and globally accepted. It may identify subclinical, microscopic peritoneal spread and thus provide valuable staging and prognostic information for chemo‐naïve ovarian cancer patients.^11^ Although the clinical utility of PWC has not been established for patients undergoing IDS, a higher risk of recurrence would be predicted for patients with positive PWC during IDS, reflecting microscopic residual disease. The aim of this retrospective study was to determine whether PWC during IDS could help predict the prognosis of patients with pelvic HGSC achieving R0 status.

## 2 MATERIALS AND METHODS

### 2.1 Patients and study design

We conducted this retrospective study in accordance with the Declaration of Helsinki. The study was approved by the institutional review board of the National Cancer Center, Japan. We followed the Reporting Recommendations for Tumor Marker Prognostic Studies of the National Cancer Institute. Between January 2007 and May 2018, 110 patients with ovarian/tubal/primary peritoneal HGSC received at least 3 cycles of platinum‐based NACT, followed by IDS at the National Cancer Center Hospital, Tokyo, Japan. All the patients achieved R0 debulking status, that is, no macroscopic residual tumor was present in the peritoneal cavity at the completion of IDS.

### 2.2 Diagnosis, treatment, and follow‐up

At least 2 gynecological pathologists reviewed all the biopsy specimens obtained from the patients at prechemotherapy to confirm the histological type of HGSC with ancillary studies such as PAX8, WT‐1, and p53 immunostaining.^12^

All the patients had received 3‐5 cycles of NACT of taxane and platinum. The patients underwent progressive disease through the cycles of NACT did not receive IDS and were thus excluded. Standard cytoreductive surgery comprising hysterectomy, bilateral salpingo‐oophorectomy, omentectomy, and lymphadenectomy was performed. To achieve optimal debulking, additional procedures such as resection of bowel, diaphragm, spleen or peritonectomy were performed. Clinical data, including the age at diagnosis, FIGO stage,^11^ extent of surgery, residual disease, serum CA 125 levels before NACT, and levels in the follow‐up period, were also gathered.

All the patients were followed every 2 months for the first 2 years after surgery and every 3‐6 months thereafter for the next 5 years or more. Relapse and progression within 6 months were defined by radiological evidence of disease recurrence or progression. The overall survival (OS) was calculated from the date of NACT initiation to the date of death, with surviving patients censored at the date of last contact. Progression free survival (PFS) was calculated from the date of NACT initiation to the date of radiological evidence of progression or death, whichever was confirmed first. No patient included in the analysis was lost to follow‐up. The median follow‐up period was 32 (5‐133) months.

### 2.3 Intraoperative peritoneal washing cytology

**Figure 1:**
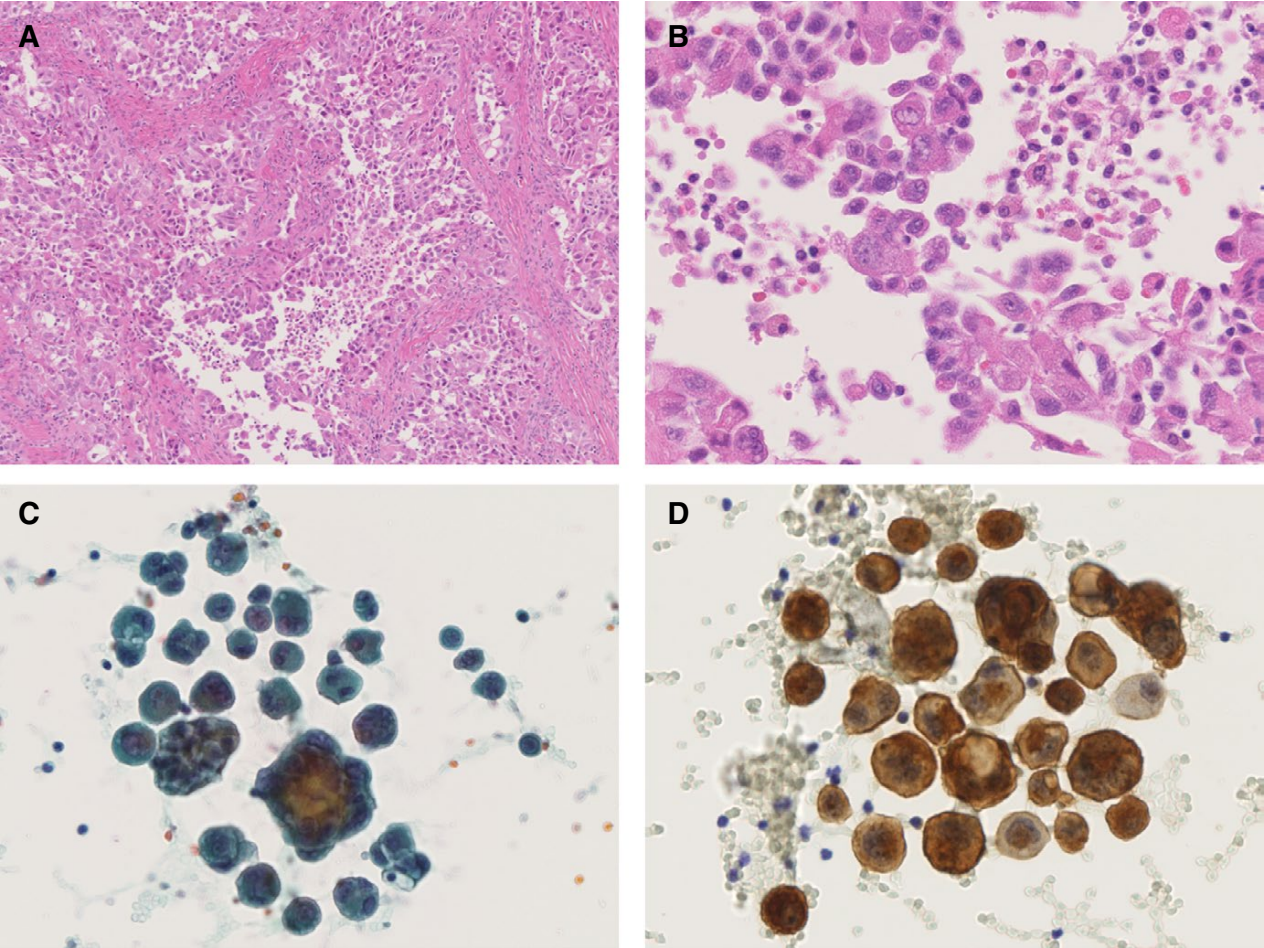
Pathological findings of a representative case with positive peritoneal washing cytology. A and B, Histological findings of primary tumor. C, Papanicolaou staining of peritoneal fluid, D, Immunocytostaining shows positivity for BerEP4, an epithelial marker

Intraoperative PWC was routinely performed for all patients who underwent IDS. During the laparotomy for IDS, ascitic fluid in the Douglas' pouch was aspirated before intraperitoneal inspection. If fluid was absent, a peritoneal wash of the Douglas' pouch was conducted with 50 mL of warmed saline and then the fluid was used for PWC. The fluids were centrifuged, and the cell pellets were evaluated microscopically by certified screeners and pathologists using Papanicolaou, Giemsa, and Alcian‐blue staining. Immunocytochemistry of BerEP4 and CEA were also performed for all the cases (Figure 1). All the cytopathological assessments were performed by certified pathologists. The results of cytological examination were classified as negative, indeterminate, suspicious, or definitive for malignancy. A definite malignancy was deemed when there were abnormal mitotic figures as well as cells with variable sizes, hyperchromatic, and irregularly shaped nuclei on Papanicolaou and Giemsa staining. In specimens sharing a part of cytological features of definite malignancy, the specimens were classified into the suspicious category. For the purpose of this study, when suspicious or definitive findings of malignancy were observed, the specimen was classified as positive for malignancy; all other findings were deemed as negative for malignancy.

### 2.4 Statistical analysis

Patient characteristics were compared by the chi‐square test or Student's *t* test. Survival curves were constructed by the Kaplan‐Meier method and compared using the log‐rank test. A Cox proportional hazards model with stepwise selection was used for multivariate analysis. Statistical differences were considered significant when the 2‐sided *P*‐value was <0.05. All statistical analyses and graphic presentations were performed with EZR^7^ (version 1.37; Saitama Medical Center, Jichi Medical University, Saitama, Japan), which is a graphical user interface for R (version 3.4.1; The R Foundation for Statistical Computing, Vienna, Austria).^13^

## 3 RESULTS

### 3.1 Patient characteristics

The baseline characteristics of the 110 patients in this study are described in Table 1. The median age of the patients was 60 years (range, 36‐87 years). Stage IV disease was noted in 48 (44%) and 110 patients (100%) had achieved complete cytoreduction with no macroscopic residual disease. Seventy‐three patients (66.4%) had received 3 cycles, 30 (27.3%) had received 4 cycles, and 7 (6.4%) had received 5 to 11 cycles of NACT prior to surgery. Of the 110 patients, 65 and 45 were PWC‐positive and PWC‐negative, respectively. The reduced serum CA 125 level as a response to NACT varied from 22.6% to 99.9%. Disease relapse was observed in 70 out of 110 (63.6%) patients and 34 (31.0%) patients had died by the endpoint of this study. The median follow‐up period for the entire study cohort was 32 months (range, 5 to 133 months). Microscopic or macroscopic residual peritoneal dissemination in the resected specimen correlated with a high proportion of PWC‐positive cases. The other clinicopathological characteristics were comparable and were almost evenly distributed in the PWC‐positive and PWC‐negative groups.

**Table 1:** Patient characteristics

Characteristic	All	PWC‐positive	PWC‐negative	*P*‐value
Number of patients	110 (100)	65 (100)	45 (100)	
Age, median [range] (y)	60 [36‐87]	59 [39‐87]	62 [36‐82]	0.25^a^
Histology, HGSC	110 (100)	65 (100)	45 (100)	
Stage (FIGO2014)				0.48^b^
II	1 (1)	0 (0)	1 (2)	
III	61 (55)	35 (54)	26 (58)	
IV	48 (44)	30 (46)	18 (40)	
CA125 level pre‐NACT	2,325 ± 318	2,969 ± 488	1,395 ± 279	0.01^a^
CA125 level post‐IDS	32 ± 3.8	36 ± 6.0	27 ± 3.5	0.25^a^
CA125 decline rate (%)	94.0 ± 1.0	95.0 ± 1.4	92.7 ± 1.5	0.27^a^
Taxane + platinum regimen	110 (100)	65 (100)	45 (100)	
NACT cycle	3.6 ± 0.12	3.4 ± 0.11	3.8 ± 0.24	0.16^a^
3 cycles	73 (66)	45 (69)	28 (62)	0.55^b^
4 cycles	30 (27)	17 (26)	13 (29)	
≥5 cycles	7 (6)	3 (5)	4 (9)	
RD status (R0)	110 (100)	65 (100)	45 (100)	
Materials for cytology				0.24^b^
Ascitic fluid	45 (41)	30 (46)	15 (33)	
Washings with saline	65 (59)	35 (54)	30 (67)	
Residual peritoneal dissemination in the resected specimens				<0.01^b^
Absent	7 (6)	0 (0)	7 (16)	
Microscopic lesion	15 (14)	5 (8)	10 (22)	
Macroscopic lesion	88 (80)	60 (92)	28 (62)	
Death within 30 days of surgery	0 (0)	0 (0)	0 (0)	
Follow‐up duration, median [range] (month)	32 [5‐133]	32 [5‐133]	31 [7‐114]	0.61^a^

Note

Data are shown as number (%) or mean ± standard error (range).

Abbreviations: CA125, cancer antigen 125; EL, exploratory laparotomy; IDS, interval debulking surgery; NACT, neoadjuvant chemotherapy; PWC, peritoneal washing cytology.

a Student‐*t* test.

b Fisher's exact test.

### 3.2 Survival analysis

**Figure 2:**
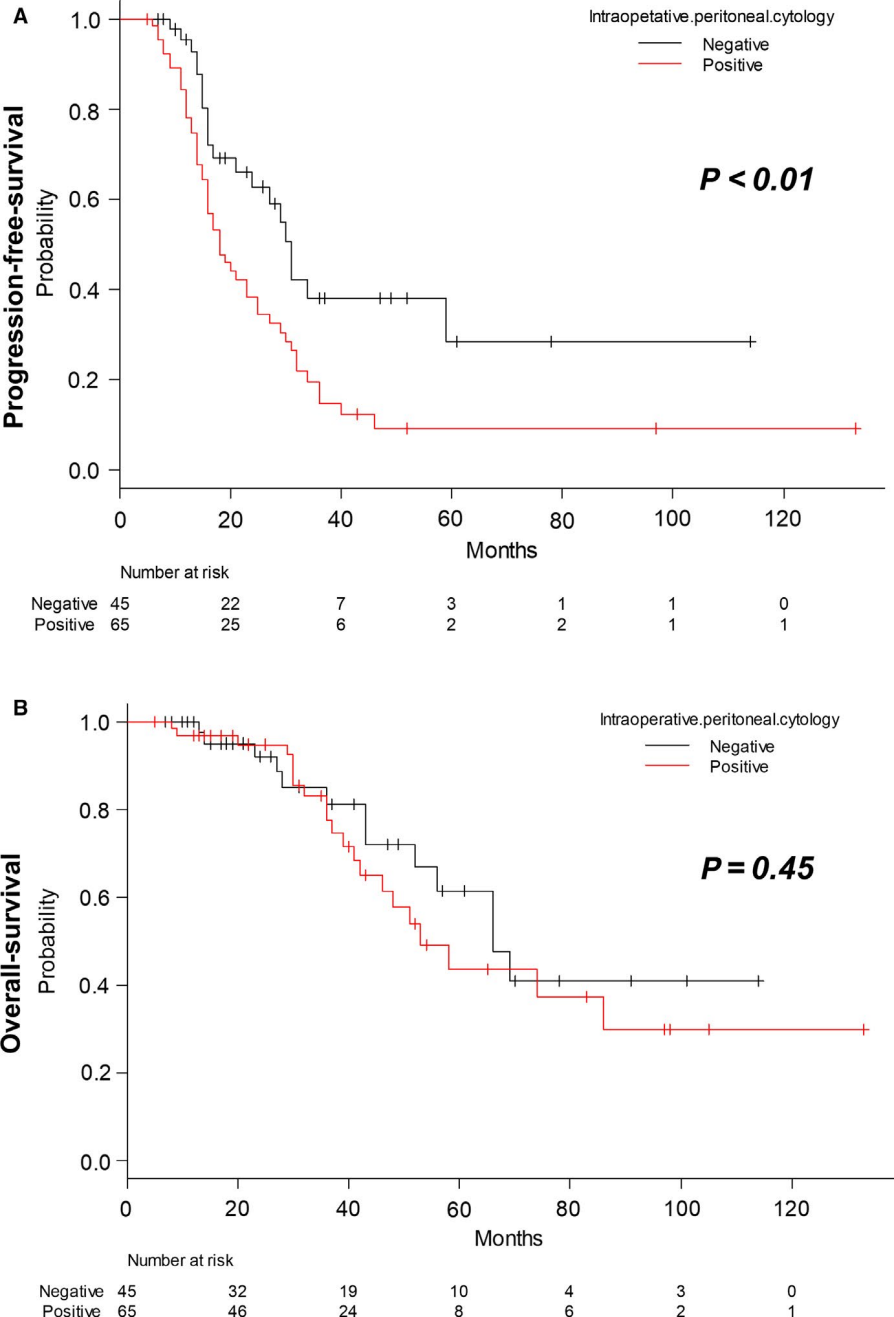
Survival analysis of patients with pelvic high‐grade serous carcinoma achieving R0 status. A, Progression‐free‐survival curves using the Kaplan‐Meier method according to intraoperative peritoneal cytology and log‐rank *P*‐values. B, Overall‐survival curves using the Kaplan‐Meier method according to intraoperative peritoneal cytology and log‐rank *P*‐values

The median PFS for the entire cohort was 17 months (range, 5‐133 months). The median PFS for patients with positive PWC was 16 months, and that of PWC‐negative patients was 19 months. Table 2 shows an increased risk of progression in patients with positive PWC (HR 2.04, 95% CI 1.22‐3.41, *P* < 0.01). Increased risk of progression was observed in both univariate and multivariate analyses (HR 2.28; 95% CI 1.35‐3.84, *P *<* *0.01). Furthermore, patients who were elderly (>60 years old) showed significantly increased risk of recurrence on the univariate analysis, whereas stage IV disease did not reach statistical significance. Figure 2A,B demonstrate the Kaplan‐Meier survival curves for PFS and OS by PWC status.

**Table 2:** Univariate and multivariate analyses for survival outcome

Factor	N	Univariate analysis				Multivariate analysis			
		5‐year PFS		5‐year OS		5‐year PFS		5‐year OS	
		HR (95% CI)	*P*	HR (95% CI)	*P*	HR (95% CI)	*P*	HR (95% CI)	*P*
Age									
≥60 (vs < 60)	58	1.65 (1.02‐2.66)	0.04	1.67 (0.83‐3.34)	0.15	1.93 (1.18‐3.17)	0.009	1.82 (0.90‐3.67)	0.09
Stage (FIGO2014)									
IV (vs II or III)	48	1.21 (0.75‐1.94)	0.44	1.71 (0.87‐3.36)	0.12	1.21 (0.75‐1.94)	0.44	1.81 (0.91‐3.61)	0.09
PWC									
Positive (vs negative)	65	2.04 (1.22‐3.41)	0.007	1.30 (0.65‐2.61)	0.46	2.28 (1.35‐3.84)	0.002	1.22 (0.61‐2.47)	0.57

Abbreviations: CI, confidence interval; HR, hazard ratio; OS, overall survival; PFS, Progression‐free survival; PWC, peritoneal washing cytology.

The results of the univariate and multivariate analyses for OS are also shown in Table 2. Median overall survival for the entire study group was 32 months (range, 5‐133 months). The median overall survival for patients with positive PWC was 32 months and for patients with PWC‐negative was 31 months. Factors such as age (>60 years), FIGO stage, and PWC status were not significant in the prediction of reduced OS on univariate and multivariate analyses.

Because residual peritoneal dissemination in the resected specimen correlated with a high proportion of PWC‐positive cases, we compared PFS between the PWC‐positive and PWC‐negative groups in only cases with any residual peritoneal disease (n = 103) or only cases with macroscopic lesions (n = 88). In both comparisons, PFS of the PWC‐positive group was found to be significantly shorter than of the PWC‐negative group (median PFS: 18 months vs 29 months, *P* = 0.046; median PFS: 18 months vs 31 months, *P* < 0.01) (Supporting Information Figure S1).

## 4 DISCUSSION

This study revealed that positive PWC during IDS had worse prognostic impact on patients with pelvic HGSC without macroscopic residual tumor. PWC may be a simple and effective method to detect clinically significant microscopic residual disease in patients with pelvic HGSC.

A few previous studies have reported on the prognostic impact of PWC during IDS in patients with ovarian cancer. Nagasaka et al demonstrated that positive‐PWC at interval surgery, including IDS, predicted significantly shorter PFS (HR 2.36, 95% CI 1.08‐5.29) in the 50 patients with stage IIIc ovarian cancer including the 37 HGSC.^14^ However, this study included 33 patients who underwent primary debulking surgery and chemotherapy and only 17 patients who underwent IDS. Furthermore, only 64% of patients achieved R0 status. In another study, Iura et al reported that negative PWC during IDS was indicative of increased overall survival (HR 0.28, 95% CI 0.089‐0.860) in 50 patients with advanced ovarian cancer, including the 28 HGSC subtype.^15^ Multivariate analysis revealed PWC‐negative, optimal debulking status (<1 cm), whereas the serous histology and number of NACT cycles were independent prognostic factors for OS. However, PWC‐negative had no prognostic impact on PFS. Unfortunately, both studies had small sample sizes, and included different histological subtypes and different debulking statuses. Moreover, these reports did not describe detailed methods of PWC such as additional staining or classification of the results. These limitations made it difficult to perform a direct comparison of these results and interpret the true prognostic impact of positive PWC on HGSC patients with R0 status. Nonetheless, positive‐PWC during surgery after NACT seems to have a consistently negative prognostic impact.

The poor prognosis of positive PWC seems to reflect the presence of microscopic residual cancer cells in the peritoneal cavity. PWC has reportedly been a sensitive indicator of ovarian surface involvement and peritoneal dissemination of ovarian tumors.^9^, ^10^ For chemo‐naïve patients with ovarian cancer, PWC reflects the microscopic peritoneal spread and thus provides valuable staging and prognostic information.^11^ The present study demonstrated that patients with positive PWC had significantly reduced PFS but had no significant impact on the OS. This may be explained by the fact that treatment after disease progression considerably varied among the patients.

The strengths of this study are the inclusion of patients with uniform characteristics, such as histological type (HGSC), debulking status (R0 status), patients receiving IDS, and a relatively large sample size (over 100 cases) compared to previous reports. Furthermore, the results of PWC were confirmed by 2 certified screeners and at least 2 pathologists by using special stains and immunocytological staining. This careful examination contributed to increased detection rate of positive‐PWC in this study. Compared to the positive‐PWC rate in patients with R0 status (28%‐39%) reported by previous studies,^14^, ^15^ this study showed a PWC‐positive rate of 59%.

Using PWC, it was possible to identify those with microscopic residual disease and reduced PFS; therefore, more intensive therapy or a chemotherapy regimen different from neoadjuvant chemotherapy may be considered for improving clinical outcomes. Furthermore, microscopic residual tumor cells after taxane and platinum chemotherapy would have resistance to these cytotoxic agents; hence, these residual cancer cells may offer opportunities to characterize the residual tumor or assess drug sensitivity to another agent after IDS.^16^, ^17^

Intraoperative PWC may be simpler and more easy‐to‐implement than histopathological assessment of chemotherapy response by tissue sections. Recently, the International Collaboration on Cancer reporting (ICCR) issued guidelines on the reporting of pelvic HGSC^18^ and recommended the inclusion of a 3‐point scoring system, namely the Chemotherapy Response Score (CRS) that was developed by Böhm et al, as standard for assessment of tumor regression following NACT.^18^, ^19^ For improving inter‐observer reproducibility of CRS, the training web site has been launched; furthermore, various external validation studies have confirmed the prognostic impact of CRS.^20^, ^21^, ^22^, ^23^ However, assessment of CRS requires an appropriate number of sampling from the omentum and training of the scoring practice.^24^, ^25^ In contrast, PWC has been already utilized in the daily practice of gynecologic surgery and needs no additional training or sampling. In addition, the qualitative difference between CRS and PWC should be recognized; thus, CRS evaluates tissue response in the resected tumor tissue while PWC reflects a part of residual tumor cells remaining in the peritoneal cavity.

The limitations of our study are that it was retrospective in design and was performed in a single institution. Therefore, a large‐scale multi‐institutional prospective trial to validate the efficacy of PWC during IDS is warranted.

In conclusion, it can be deduced that a positive PWC during IDS predicts earlier disease recurrence in patients with pelvic HGSC, even in those achieving R0 status. As performing PWC during IDS becomes standard practice, prospective validation should be conducted in the future.

## CONFLICT OF INTEREST

The authors declare that they have no conflict of interest.
