# Supplementary figures and images for: Prognostic impact of intraoperative peritoneal cytology in interval debulking surgery for pelvic high‐grade serous carcinoma
Source: Cancer Med. 2019 Jun 26;8(10):4598–604. doi: 10.1002/cam4.2377 (PMC6712449; doi:10.1002/cam4.2377)

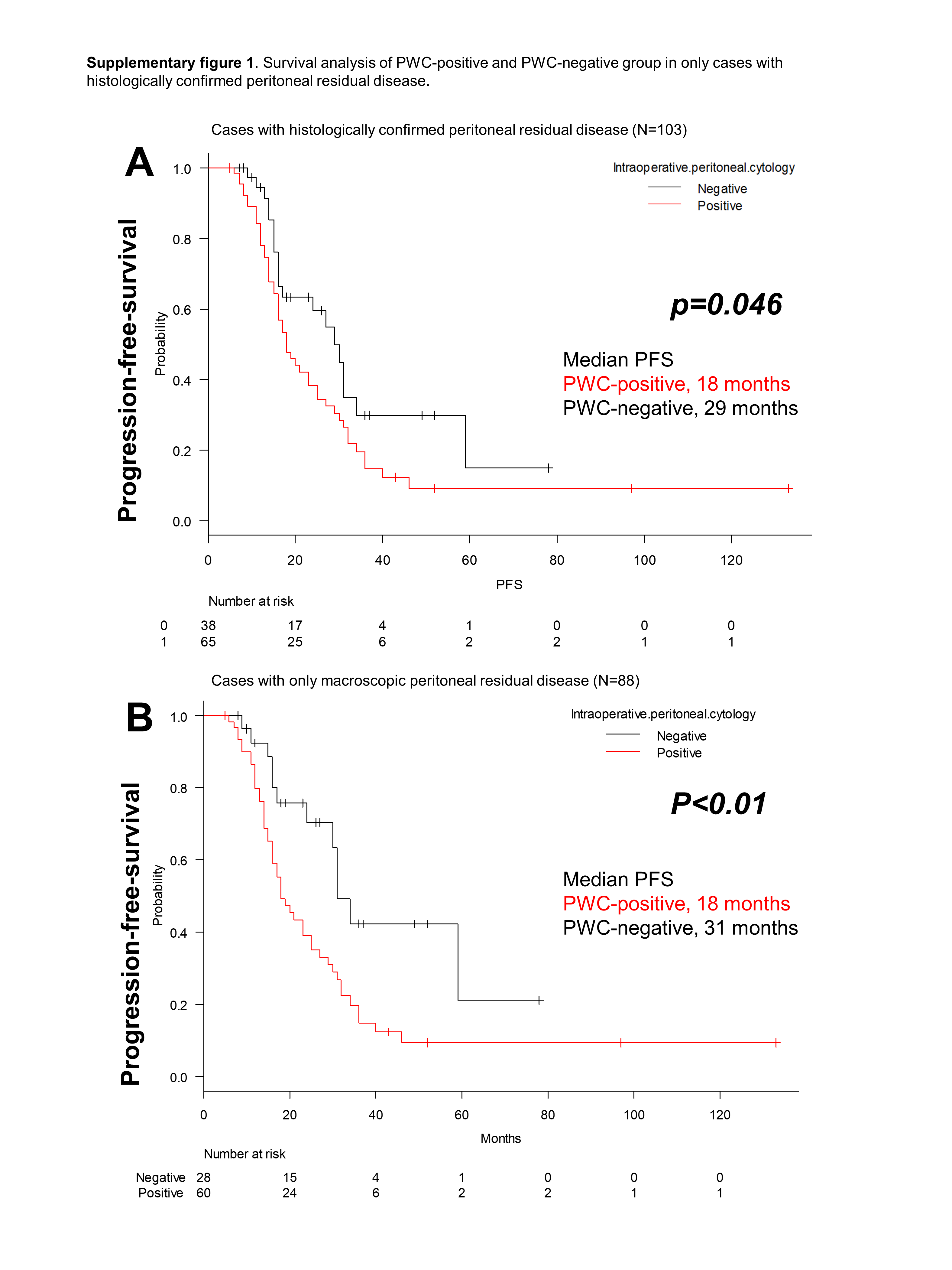

Supplement: Supplementary file 1 [file CAM4-8-4598-s001.tif]
